# Dysmenorrhoea in Different Settings: Are the Rural and Urban Adolescent Girls Perceiving and Managing the Dysmenorrhoea Problem Differently?

**DOI:** 10.4103/0970-0218.43231

**Published:** 2008-10

**Authors:** Atchuta Kameswararao Avasarala, Saibharghavi Panchangam

**Affiliations:** Department of Community Medicine, Prathima Institute of Medical Sciences, Karimanagar, Andhra Pradesh 505417, India

**Keywords:** Dysmenorrhoea in different settings, Karimnagar district, management differences, social losses, urban-rural variations

## Abstract

**Context:**

It is well-known that every health problem, not only presents itself with different epidemiological profiles in different population settings, but is also perceived and managed differently. Having knowledge of these variations in its presentations and perceptions in different population settings, for example, in urban and rural settings, will be useful for its successful management.

**Aim:**

To study differences in epidemiological profiles, perceptions, socio economic losses, and quality-of-life losses and management of dysmenorrhoea in different settings for effective management.

**Design and Setting:**

A comparative cross-sectional study among adolescent school girls (101 girls in urban areas and 79 girls in rural areas) in the district of Karimnagar.

**Materials and Methods:**

A cross-sectional survey using a pretested questionnaire was conducted among 180 adolescent girls in urban and rural settings.

**Statistical Analyses Used:**

Proportions and X^2^ test.

**Results:**

The prevalence of dysmenorrhoea is 54% (53% in girls in urban areas and 56% in girls in rural areas) (X^2^ _df_ = 0.1, *P* = 0.05). Sickness absenteeism (28–48%), socio economic losses, and perceived quality of life losses are more prevalent among girls in urban areas than in girls in rural areas. Girls in rural areas resort to physical labor and other natural methods to obtain relief while the girls in urban areas are mainly depending on medications.

**Conclusions:**

Dysmenorrhoea can also be managed effectively by natural methods without resorting to medicines, provided one is psychologically prepared to face it without anxiety.

## Introduction

Primary dysmenorrhoea is defined as painful menses in women with normal pelvic anatomy, usually beginning during adolescence. It is characterized by crampy pelvic pain beginning shortly before or at the onset of menses and lasting 1 to 3 days.(1) It not only disturbs their routine but also causes humiliating suffering. It is a common cause for sickness absenteeism from classes and work by the female student community.(1) It is a public health problem with its high prevalence,(1,2) suffering, and considerable economic losses. Most of the studies on dysmenorrhoea have emphasized mainly on the drug management, while only a few stressed on cultural practices(3) and the perceptions in different settings. It is well-known that every health problem not only presents itself with different epidemiological profiles in different population settings but is also perceived and managed differently. The knowledge of these variations in its presentations and perceptions in different population settings, for example, in urban and rural settings, will be useful for its successful management.

Hence, this study on dysmenorrhoea is attempting to learn about the differences in the epidemiological profiles, perceptions of the problem, social losses, quality-of-life losses and the management differences among adolescent girls in urban and rural settings.

## Subjects and Methods

A female doctor and a female medico-social worker were trained for this survey. A total of 101 girls from urban schools and families in Rampur and 79 girls from a rural school in the village of Nagunur were selected by random sampling for this study. Open-ended questionnaires relating to age, sex, the literacy status of the mother, nutritional status, duration of dysmenorrhoea, periodicity of menstrual cycles, duration of the menstrual cycle, amount of blood loss, time of onset of pain, character of pain, causes of dysmenorrhoea and pain relieving measures, perceptions and attitudes, management techniques, social and economic losses, and quality-of-life losses were completed. The questionnaires were pre-tested among 20 students in urban and rural areas.

The following criteria are used to define dysmenorrhoea:(1)

- Onset of pain within 6–12 hours after menarche.
- Lower abdominal or pelvic pain associated with onset of menses and lasting for 8–72 hours.
- Lower back pain during menses.
- Medial or anterior thigh pain.
- Menstrual pain with associated features like headache, diarrhea, nausea, vomiting.

The survey was completed and data was analyzed for the results.

## Results

### Study population

There were 180 respondents between the ages of 19 to 25 years old. The average age at menarche was 12 to 13 years old; the mean duration of menstrual flow was 1–5 days; and the menstrual cycle length varied between 28–35 days. A total of 169 (93.8%) of the respondents had a regular menstrual cycle, while 11 respondents (6.2%) had irregular menses.

### Dysmenorrhoea

The overall prevalence of dysmenorrhoea was 54% (52.5% in the urban areas and 55.7% in the rural areas, respectively). The difference between girls in urban areas versus girls in rural areas is not significant (X^2^_df1_ =0.1, *P*=>0.05) [Table 1].

**Table 1:** Urban–rural distribution of dysmenorrhoea

Dysmenorrhoea	Urban group (%) n = 101	Rural group (%) n = 79	Total (%) n = 180
Dysm+ve	53 (52.5)	44 (55.7)	97 (53.89)
Dysm-ve	47 (47.5)	35 (44.3)	83 (46.11)

The three leading risk factors, in order, are sensitive and anxious personalities, a positive family history, and stress and are seen more often in girls in urban areas than in girls in rural areas [Table 2]. Only the stress factor is highly significant (X^2^ =7.68, *P*<0.01) [Table 3]. While heavy flow is reported (53%) in the urban group, scanty flow is observed (46%) in the rural group. Premenstrual tension was reported by 37% of girls in the urban group compared with 12% of girls in the rural group. Pain is mainly intermittent, commencing with the onset of menses in both groups. Forty-two percent of sufferers in the urban group belonged to middle-class families while 90% of the rural girls were from poorer backgrounds. The mother's literacy rate was seen more with girls in the urban group (90%) than in girls in the rural group (2%). Dysmenorrhoea among girls from urban areas was not dependent upon literacy status of their mothers. The suffering was also independent of nutritional status in the urban setting, while it is related in the rural setting (52%).

**Table 2:** Dysmenorrhoea and its risk factors

Risk factors	Urban group (%) n = 53	Rural group (%) n = 44	Total (%) n = 97
Positive family history	35 (66)	22 (50)	57 (58.7)
Sensitive personality	41 (77.3)	26 (59)	67 (68.8)
Stress	32 (60)	11 (25)	43 (45)

**Table 3:** Stress and dysmenorrhoea

Stress factor	Urban group (%) n = 53	Rural group (%) n = 44	Total (%) n = 97
Stress present	32 (60.3)	11 (25)	43 (44)
Stress free	21 (39.7)	33 (75)	54 (55.7)

Sickness absenteeism is frequent in girls in urban areas (36–72%) while it is rare (18–48%) in girls in the rural areas [Table 4]. Quality-of-life losses are seen more with girls in urban areas than their rural counterparts [Table 5]. While girls in rural areas indulged in physical labor to get relief, the girls in urban areas were dependant on self-medication and rest.

**Table 4:** Sickness absenteeism due to dysmenorrhoea

Sickness absenteeism in 2007	Urban group (%) n = 53	Rural group (%) n = 44	Total (%) n = 97
Absent from classes	38 (71.6)	9 (20.5)	47 (48.5)
Absent from exams	19 (35.8)	8 (18)	27 (27.8)
Assignment not completed	28 (52.8)	6 (13.6)	34 (35)
Reprimanded by teachers	36 (67.9)	21 (47.7)	97 (100)

**Table 5:** Quality-of-life losses due to dysmenorrhoea

Life facet	Urban group (%) n = 53	Rural group (%) n = 44	Total (%) n = 97
Poor general adoption	31 (58.5)	17 (38.6)	48 (49.48)
Loss of physical independence	29 (54.7)	7 (15.9)	40 (42.42)
Poor work satisfaction	35 (66)	15 (34)	50 (51.54)
Personal relationships not good	25 (47)	11 (25)	36 (37.11)
Social integration not good	27 (50.1)	6 (13.6)	33 (34.02)
Physical activity not good	38 (71.7)	11 (25)	49 (50.51)
Leisure activities not good	22 (41.5)	12 (27)	34 (35.05)

## Discussion

### Prevalence

The prevalence of dysmenorrhoea among adolescent girls was 54% in this study, which is almost the same as reported by other Indian and western studies.(4,5) Old literature from the Middle East and Europe is rippled with studies showing high prevalence. This means almost more than half of the adolescent girls throughout the world suffer from dysmenorrhoea and need attention. In this study, prevalence of dysmenorrhoea appears to be little more (3%) in rural girls.

**Epidemiological differences:** Dysmenorrhoea is found to be more of familial nature among girls in urban areas in this study, even though they are better placed, nourished, and educated than their rural counterparts. These are not related to dysmenorrhoea as was also reported by French.(5) Premenstrual tension with heavy menstrual flow, stress, socio-economic losses, and quality-of-life losses are seen more with girls in urban areas than girls in rural areas. This seems to be due to their sensitive and anxious personality and high levels of stress. A stress factor was highly associated with dysmenorrhoea in this study as was also reported by Wang *et al.*(6) Good nutrition is not protecting the girls in urban areas but poor nutrition is affecting the girls in rural areas. This suggests that stress is the principal factor rather than nutritional status.

**Differences in perceptions:** The perception of dysmenorrhoea was entirely different in the two study groups. The girls in urban areas are affected more seriously and more often resort to treatment. The girls in rural areas consider it as a regular, unavoidable problem, and manage it by endurance only(7) and do not panic. This attitude of low tolerance among girls in urban areas leads to more premenstrual suffering, heavy flow, socioeconomic losses, and loss of qualitative life due to dysmenorrhoea. Though their mothers are more educated, they are not contributing much to their daughters regarding development of proper attitude to manage dysmenorrhoea. On the other hand, girls in rural areas who are tackling this problem with endurance are not having any major losses of such type. Probably, though illiterates, their mothers are able to develop a proper attitude in their children to face dysmenorrhoea.

**Differences in socio-economic losses:** Girls in urban areas are not only suffering more but also missing their classes and work. Weisman *et al.*(8) observed similar findings in their study. Moreover, millions of dollars are being wasted due to sickness absenteeism because of dysmenorrhoea in the U.S.A. as shown by Bergsjo.(9)

**Differences in quality-of-life losses:** The quality-of-life during dysmenorrhoea is comparatively poor among girls in urban areas: loss of physical motility and work, relationships, social mingling, and leisure opportunities also suffer. This clearly indicates that dysmenorrhoea is disturbing their life more when compared with the lives of girls in rural areas. The restricted activity seen in this study is also found in other studies like Adeyami *et al.* Life is not that bad with girls living in rural areas. They are not losing life chances or classes to the extent lost by girls living in urban areas and moreover they are overworking during that period of dysmenorrhoea and having better work satisfaction than their counterparts in urban areas.

**Management differences:** The girls in urban areas are nervous, sensitive, and cannot endure dysmenorrhoea and they have resorted to self-medication even without the advice of a doctor as also shown by El Gilany *et al.*(10) They are simply following the usual prescription for dysmenorrhoea, which is “Take rest and pain killers.” They are trying NSAIDs, Paracetomol, Vitamin E supplements, and oral contraceptive pills for the relief of premenstrual tension and pain.(11–14) In addition, they resort to rest and escaping work, which results in more sickness absenteeism. On the contrary, the girls in the rural areas are adapting to the situation by endurance and managing the problem without drugs to a large extent. They belittle the suffering by indulging in work and diverting their attention or by using natural methods such as cold baths, home remedies like Fenugreek seed powder in water, etc. and avoid taking rest.(15) Dissemination of this information about endurance of dysmenorrhoea among the adolescent girls and their mothers may help change the attitude towards management of dysmenorrhoea. Teenage clubs may be involved in conducting stress releasing programs.

To conclude, this study confirmed that dysmenorrhoea is more of a psychosomatic disorder and it can be better managed by mental preparation and by simply immersing in work without resorting to drugs or losing any measure of quality of life. Studies like this on the differences in profiles in different settings will help the health planner to plan for proper and effective interventions suitable to those particular settings.

## References

[1] Adeyemi AS, Adekanle DA (2007). Management of dysmenorrhoea among medical students. Int J Gynecol Obstet.

[2] Banikarim C, Chacko MR, Kelder SH (2000). Prevalence and impact of dysmenorrhoea on Hispanic female adolescents. Arch Pediat Adolesc Med.

[3] Burnett MA, Antao V, Black A, Feldman K, Grenville A, Lea R (2005). Prevalence of primary dysmenorrhea in Canada. J Obstet Gynaecol Can.

[4] Nair P, Grover VL, Kannan AT (2007). Awareness and practices of menstruation and pubertal changes amongst unmarried female adolescents in a rural area of East Delhi. Indian J Community Med.

[5] French L (2005). Study on Dysmenorrhoea, American academy of family Physicians. J Postgrad.

[6] Wang L, Wang X, Wang W, Chen C, Ronnennberg AG, Guang W (2004). Stress and dysmenorrhoea: A population based prospective study. Occup Environ Med.

[7] Goldstein-Ferber S, Granot M (2006). The association between somatization and perceived ability: roles in dysmenorrhoea among Israeli Arab adolescents. Psychoso Med.

[8] Weissmen AM, Hartz AJ, Hansen MD, Johnson SR (2004). The natural history of primary dysmenorrhoea: A longitudinal study. BJOG.

[9] Bergsjo P (1979). Socioeconomic implications of dysmenorrhoea. Acta Obstet Gynecol Scand.

[10] El-Gilany AH, Badawi K, El-Fedawy S (2005). Epidemiology of dysmenorrhoea among adolescent students in Mansoura, Egypt. East Meditarr Health J.

[11] Tzafettas J (2006). Painful menstruation. Pediatr Endocrinol Rev.

[12] Marjoribanks J, Proctor ML, Farquar C (2003). Nonsteroidal anti-inflammatory drugs for primary dysmenorrhoea. Cochrane Database Syst Rev.

[13] Ziaei S, Zakeri M, Kazemnejad A (2005). A randomized controlled trial of vitamin E in the treatment of primary dysmenorrhoea. Br J Obstet Gynaecol.

[14] Davis AR, Westhoff C, O'Connell K, Gallagher N (2005). Oral contraceptives for dysmenorrhoea in adolescent girls: A randomized trial. Obstet Gynecol.

[15] Proctor M, Farquhar C (2000). Dysmenorrhoea. Clinical Evid.

